# Correction to: Incident autoimmune diseases in association with SARS-CoV-2 infection: A matched cohort study

**DOI:** 10.1007/s10067-023-06692-8

**Published:** 2023-07-05

**Authors:** Falko Tesch, Franz Ehm, Annika Vivirito, Danny Wende, Manuel Batram, Friedrich Loser, Simone Menzer, Josephine Jacob, Martin Roessler, Martin Seifert, Barbara Kind, Christina König, Claudia Schulte, Tilo Buschmann, Dagmar Hertle, Pedro Ballesteros, Stefan Baßler, Barbara Bertele, Thomas Bitterer, Cordula Riederer, Franziska Sobik, Lukas Reitzle, Christa Scheidt-Nave, Jochen Schmitt

**Affiliations:** 1 Center for Evidence-Based Healthcare (ZEGV), University Hospital and Faculty of Medicine Carl Gustav Carus at TU Dresden, Fetscherstraße 74, 01307 Dresden, Germany; 2 InGef - Institute for Applied Health Research Berlin GmbH, Berlin, Germany; 3 BARMER Institut für Gesundheitssystemforschung (bifg), Berlin, Germany; 4 Vandage GmbH, Bielefeld, Germany; 5 Techniker Krankenkasse, Hamburg, Germany; 6 IKK classic, Dresden, Germany; 7 AOK PLUS, Dresden, Germany; 8 DAK-Gesundheit, Hamburg, Germany; 9 Robert Koch Institute, Berlin, Germany

**Correction to: Clinical Rheumatology** 10.1007/s10067-023-06670-0

The Figs. 1 and 2 images of the original published version of the above article were interchanged. Now the Figures have been corrected, and the legends remain unchanged as follows:

**Fig. 1:**
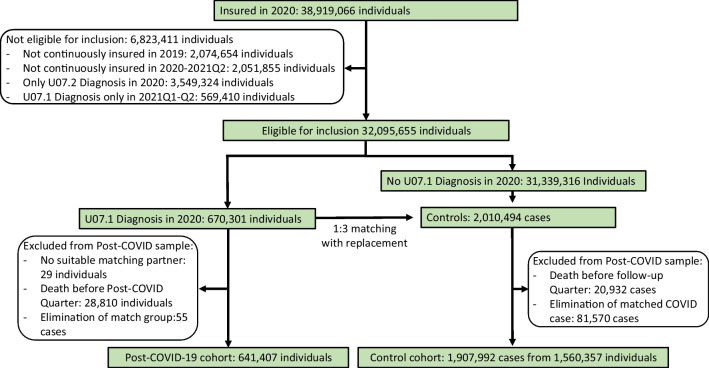
Flowchart for the selection of the COVID-19 and control groups

**Fig. 2:**
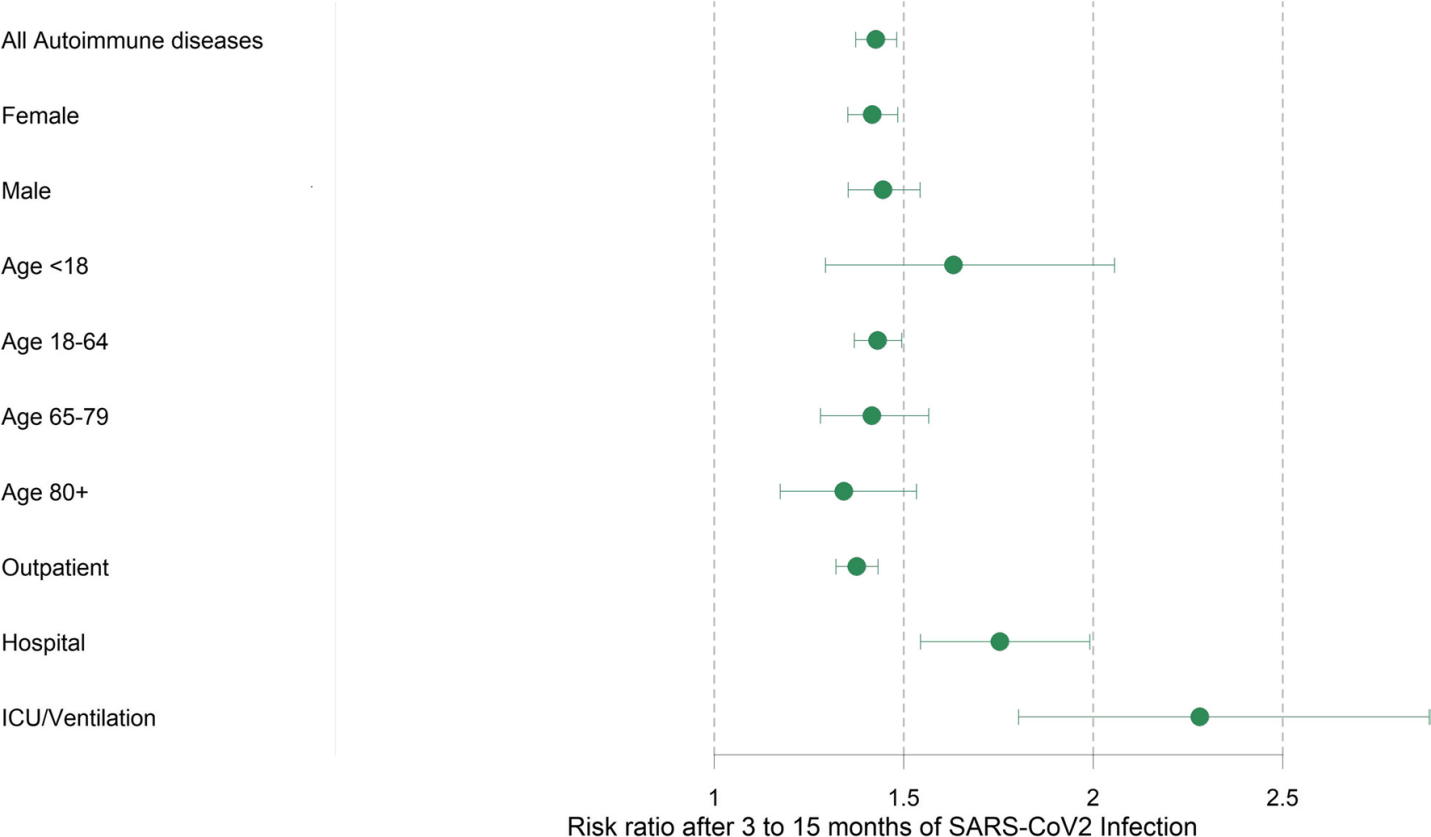
Forest plot comparing incident rate ratios for any first-onset autoimmune disease 3 to 15 months after SARS-CoV-2 infection by subgroup. The severity of COVID-19 was operationalized as only outpatient care, usual hospital care, and ICU/ventilation-intensive care unit and mechanical ventilation

